# New prognostic system specific for epidermal growth factor receptor-mutated lung cancer brain metastasis

**DOI:** 10.3389/fonc.2023.1093084

**Published:** 2023-03-20

**Authors:** Li-Hua Zhu, Xing-Wen Fan, Lu Sun, Ting-ting Ni, Ya-qi Li, Chao-Yang Wu, Kai-Liang Wu

**Affiliations:** ^1^ Department of Radiation Oncology, The People’s Hospital Affiliated to Jiangsu University, Zhenjiang, China; ^2^ Department of Radiation Oncology, Fudan University Shanghai Cancer Center, Shanghai, China; ^3^ Department of Oncology, Shanghai Medical College, Fudan University, Shanghai, China; ^4^ Shanghai Clinical Research Center for Radiation Oncology, Shanghai Key Laboratory of Radiation Oncology, Shanghai, China

**Keywords:** EGFR, lung cancer, brain metastases, TKI, radiotherapy

## Abstract

**Introduction:**

Brain metastases (BM) from lung cancer are heterogeneous, and accurate prognosis is required for effective treatment strategies. This study aimed to identify prognostic factors and develop a prognostic system exclusively for epidermal growth factor receptor (EGFR)-mutated lung cancer BM.

**Methods:**

In total, 173 patients with EGFR-mutated lung cancer from two hospitals who developed BM and received tyrosine kinase inhibitor (TKI) and brain radiation therapy (RT) were included. Univariate and multivariate analyses were performed to identify significant EGFR-mutated BM prognostic factors to construct a new EGFR recursive partitioning analysis (RPA) prognostic index. The predictive discrimination of five prognostic scoring systems including RPA, diagnosis-specific prognostic factors indexes (DS-GPA), basic score for brain metastases (BS-BM), lung cancer using molecular markers (lung-mol GPA) and EGFR-RPA were analyzed using log-rank test, concordance index (C-index), and receiver operating characteristic curve (ROC). The potential predictive factors in the multivariable analysis to construct a prognostic index included Karnofsky performance status, BM at initial lung cancer diagnosis, BM progression after TKI, EGFR mutation type, uncontrolled primary tumors, and number of BM.

**Results and discussion:**

In the log-rank test, indices of RPA, DS-GPA, lung-mol GPA, BS-BM, and EGFR-RPA were all significant predictors of overall survival (OS) (*p* ≤ 0.05). The C-indices of each prognostic score were 0.603, 0.569, 0.613, 0.595, and 0.671, respectively; The area under the curve (AUC) values predicting 1-year OS were 0.565 (*p*=0.215), 0.572 (*p*=0.174), 0.641 (*p*=0.007), 0.585 (*p*=0.106), and 0.781 (*p*=0.000), respectively. Furthermore, EGFR-RPA performed better in terms of calibration than other prognostic indices.BM progression after TKI and EGFR mutation type were specific prognostic factors for EGFR-mutated lung cancer BM. EGFR-RPA was more precise than other models, and useful for personal treatment.

## 1 Introduction

Lung cancer remains the most common cause of cancer and cancer-related mortality worldwide (1),Brain metastases (BM) are common in patients with lung cancer, 20-25% of non-small-cell lung cancer (NSCLC) patients are estimated to have BM at initial diagnosis (2, 3). Around 40- 60% of the patients diagnosed with NSCLC develop BM during the course of their disease, and this cumulative risk increases up to 70% in patients with epidermal growth factor receptor (EGFR) mutation (4). Mutations that constitutively activated the EGFR kinase domain are present in 10-15% of patients with lung adenocarcinoma in North America and up to 60% of patients in Asia (5). In the past, survival after the diagnosis of BM in NSCLC patients was uniformly poor, and its management was futile (6, 7). However, with advances in systemic treatment and technology, including molecularly targeted therapies and stereotactic radiosurgery (SRS), survival from BM has improved (8). Extensive efforts have focused on predicting outcomes for the considerable heterogeneity of patients with BM. An accurate and easy diagnosis-specific tool for clinicians is urgently required to improve their ability to assess patient prognosis and create clinical risk groups for informing treatment or patient stratification by disease severity in clinical trials.

Considerable research efforts have focused on predicting outcomes for the extremely heterogeneous population of patients with BM. Gaspar et al. (9)presented RPA prognostic system, Lorenzoni et al. (10) proposed BS-BM, and Sperduto et al. (11) developed GPA. However, these BM prognostic indices included various tumor types. Sperduto et al. (12) recognized the variability of the prognostic factors according to primary diagnosis and constructed a new prognostic index named DS-GPA. Based on the effect of gene alterations on survival in patients with lung cancer, Sperduto et al. (12)proposed lung-mol GPA that included the addition of gene status. The limitation of previous studies on BM was the inconsistency in treatment methods, especially those considering EGFR mutations. Tyrosine kinase inhibitors (TKIs) and brain radiation therapy (RT), which are the most important treatments for patients with EGFR-mutated BM. Recently, the use of TKIs for treating BM in patients who are EGFR-TKI naïve has been demonstrated to have a central nervous system (CNS) objective response rate of 91% (Osimertinib) and 68% (Gefitinib or Erlotinib) (13). Inconsistency in treatment methods may affect the construction of a BM model. Whether the existing BM indices were applicable was unknown in an era of lung cancer with targeted therapies. Therefore, in this study, only the patients with EGFR-mutated NSCLC BM who received TKIs and brain RT were included in order to reduce the risk of bias. We evaluated previous BM indices and established a new prognostic index EGFR recursive partitioning analysis (RPA) referring to the RPA model based on a reasonable combination of EGFR mutation-specific predictors.

## 2 Methods

### 2.1 Study design and patients

Patients with EGFR-mutated NSCLC who were diagnosed with BM at any point of the disease course and treated with EGFR-TKI and brain radiotherapy from January 2008 to December 2018 at the Fudan University Shanghai Cancer Center and the People’s Hospital Affiliated to Jiangsu University were identified. Since this study aimed to retrospectively evaluate prognostic factors for OS and construct a new prognostic grading system for NSCLC, the following patients were included: (i) those histologically diagnosed with lung adenocarcinoma, (ii) those who presented with EGFR mutations in the primary tumor or metastatic brain lesions, (iii) those with confirmed BM using computed tomography and (or) magnetic resonance imaging, and (iv) those who received first-generation and second- generation EGFR TKIs and brain radiotherapy, including whole brain radiation therapy (WBRT) and SRS. Patients who received EGFR-TKI for less than 1 month and patients lost to follow-up were excluded. Patient data included detailed clinical data, follow-up examination results and death dates (if applicable). Patients were followed up *via* clinic visits and telephone interviews. OS was calculated from the date of diagnosis of BM to the date of death owing to cancer or by patient censoring on the date of the last follow up. All patients were followed up until death or April 2020 (end of follow-up). The study was conducted according to the Helsinki Declaration and the study protocol was approved by the Ethics Review Board of the Fudan University Shanghai Cancer Center and the People’s Hospital Affiliated to Jiangsu University.

### 2.2 Analyses of prognostic factors

To evaluate prognostic factors for OS for EGFR-mutated lung cancer BM patients, data on the following variables were gathered for the analysis: sex, age, Karnofsky performance score (KPS) at the time of BM, stage at initial diagnosis, whether patients were symptomatic because of BM, whether there was BM progression after TKI, presence or absence of extracranial metastases concurrent with the BM, EGFR mutation site, symptoms related to BM, control of the primary tumor, number of BM, and type of RT delivered. The dates of the initial cancer diagnosis, BM diagnosis, intracranial progression, RT treatments, systemic therapy treatments, most recent follow-up, and death were also recorded. These variables were included in the univariate analysis which was performed using the Kaplan–Meier method plus the log-rank test. The variables that were significant in the univariate analysis (*p<*0.05) were evaluated for independent associations with survival in the multivariate analysis (Cox proportional hazards model).

### 2.3 Construction of a new prognostic index

By referring to the RPA scoring system (9), we established a new BM scoring system named EGFR mutation-specific RPA (EGFR-RPA) based on the multivariate analysis results. The variables significantly associated with survival (*p*<0.05) in the Cox proportional hazard analysis were incorporated in the *EGFR*-RPA.

### 2.4 Analyses and assessment of prognostic stratification

To evaluate the prognostic factors for *EGFR*-mutated NSCLC BM patients receiving *EGFR*-TKI and brain RT using the prognostic grading systems, patients were stratified according to RPA, DS-GPA, BS-BM, lung-mol GPA and *EGFR*-RPA.

### 2.5 Statistics

The Kaplan-Meier analysis was used to estimate the OS, from the date of diagnosis of BM to the date of death or last follow-up. The univariate Cox proportional hazards analysis examined the factors associated with an increased risk of death. With the significant variables obtained in the univariate analysis, multivariate Cox regression analysis was performed to determine the new model for predicting survival. Log-rank testing was used to compare the adjacent classes with OS for five prognostic indices. The AUC and C-index were used to estimate the discriminative ability with the five existing indices. All analyses were performed using SPSS version 17.0 (IBM Corporation, Chicago, IL) and R version 3.5.1. (The R Foundation, Vienna, Austria).

## 3 Results

### 3.1 Patient characteristics

From January 2008 to December 2018, a total of 173 patients were included in this retrospective study conducted in two hospitals. The process of screening eligible patients is provided in the **Supplementary Materials** (**Supplement Figure 1**). The median follow-up time for these patients was 67 months (range, 1-112 months). The median age was 57 years (range, 31-84 years). The patients were predominantly ≤70 old years (91.3%), were males (77.8%), had a KPS score ≥70 (72.3%), BM ≤3 (64.2%), had extracranial metastases (ECM) (72.8%), had symptomatic BM (59.5%), had metachronous BM (52.6%), and received upfront or concurrent WBRT or SRS (79.2%). Patients’ characteristics at baseline are shown in **Table 1**.

**Table 1:** Clinical characteristics of the 173 patients.

Variables	No.	(%)
Sex		
Female	100	57.8
Male	73	42.2
Age(years)		
<50	44	25.4
50 -<60	57	32.9
60 -<70	57	32.9
>=70	15	8.7
KPS		
<70	48	27.7
70-80	96	55.5
>=90	29	16.8
Stage at diagnosis		
I-III	51	29.5
IV	122	70.5
BM at initial diagnosis		
Synchronous	82	47.4
Metachronous	91	52.6
Extracranial metastases		
Yes	126	72.8
No	47	27.2
EGFR site		
19 deletion	90	52.0
21 mutation	72	41.6
Others	11	6.4
Symptomatic BM		
Yes	103	59.5
No	70	40.5
Control of primary tumor		
Yes	122	70.5
No	51	29.5
Numbers of BM		
1	72	41.6
2-3	39	22.5
>=4	62	35.8
EGFR-TKIs		
Gefitinib	82	47.4
Erlotinib	41	23.7
Icotinib	47	27.2
Afatinib	3	1.7
Radiotherapy technology		
WBRT	122	70.5
SRS	25	14.5
WBRT+SRS	17	9.8
Surgeon+WBRT	6	3.5
Surgeon+SRS	3	1.7
Timing of radiotherapy		
Upfront or concurrent WBRT or SRS	137	79.2
Upfront EGFR-TKI	36	20.8

KPS, Karnofsky performance status; BM, Brain metastases; EGFR, Epidermal growth factor mutation; WBRT, Whole brain radiation therapy; SRS, Stereotactic radiosurgery; TKI, Tyrosine kinase inhibitors.

### 3.2 Prognostic factors for outcomes of OS

The median OS was 30 (95% confidence interval [CI], 26-34, **Supplement Figure 2**) months. In the univariate analysis, a significantly shorter OS was observed in patients with KPS <70 (*p=*0.000), BM at initial diagnosis (*p=*0.001), BM progression after TKI (*p=*0.000), extracranial metastases (*p=*0.007), *EGFR* mutation type that was not exon 19 deletion (*p=*0.007), uncontrolled primary tumor (*p=*0.000), and number of BM >3 (*p=*0.016). In addition, we observed that the patients who underwent SRS or surgical resection with or without WBRT tended to have a longer OS than those who underwent only WBRT; however, the difference was not statistically significant (*p=* 0.063). Further, there was no significant difference observed in the patients with respect to sex, age, stage at initial diagnosis, symptomatic BM, and timing of RT. In the multivariate analysis using multiple Cox proportional hazards models, we observed that the performance status (KPS<70, *p=* 0.006), BM at the time of initial lung cancer diagnosis (*p=* 0.024), BM progression after TKI (*p=*0.000), *EGFR* mutation (*p=*0.023), uncontrolled primary tumor (*p=*0.002), and more than three BM (p=0.005) were the independent prognostic factors for OS (**Table 2**).

**Table 2:** Univariable and multivariable analyses of covariables associated with OS.

	Univariable Analysis					Multivariable Analysis			
Variable	Median OS (month)	BE	95%CI		*P*	HR	95%CI		*P*
Gender					0.115				
Male	29	2.325	24.444	33.556					
Female	31	5.086	21.031	40.969					
Age					0.192				
<70	31	2.258	26.574	35.426					
>=70	27	7.059	13.164	40.836					
KPS					0.000	1.787	1.182	2.700	0.006
<70	19	2.411	14.274	23.726					
>=70	36	3.329	29.474	42.526					
Stage at diagnosis					0.964				
I-III	30	2.188	25.711	34.289					
IV	27	13.064	1.395	52.605					
BM at initial diagnosis					0.001	0.599	0.383	0.935	0.024
Synchronous	24	4.272	17.700	30.300					
Metachronous	40	3.193	31.600	48.400					
BM progrssion after TKI					0.000	2.529	1.557	4.111	0.000
Yes	10	1.829	6.415	13.585					
No	35	3.715	27.718	42.282					
Extracranial metastases					0.007				
Yes	27	2.092	22.900	31.100					
No	47	10.344	26.726	67.274					
EGFR site					0.007	1.498	1.058	2.122	0.023
19 deletion	35	3.981	27.197	42.803					
others	17	7.707	1.895	32.105					
Symptomatic BM					0.108				
Yes	27	3.426	28.285	41.715					
No	35	3.501	20.138	33.862					
Control of primary tumor					0.000	0.530	0.352	0.797	0.002
Yes	37	4.013	29.134	44.866					
No	23	2.866	17.383	28.617					
Numbers of BM					0.016	1.689	1.170	2.437	0.005
<=3	34	4.078	26.007	41.993					
>3	17	4.354	8.466	25.534					
Radiotherapy technology					0.063				
WBRT	27	3.091	20.941	33.059					
SRS/Surgeon± WBRT	32	3.383	25.370	38.630					
Timing of radiotherapy					0.249				
Upfront or concurrentWBRT or SRS	31	2.798	25.516	36.484					
Upfront EGFR-TKI	25	7.944	9.429	40.571					

KPS, Karnofsky performance status; BM, Brain metastases; TKI, Tyrosine kinase inhibitors; EGFR, Epidermal growth factor mutation; WBRT, Whole brain radiation therapy; SRS, Stereotactic radiosurgery; CI, Confidence interval.

### 3.3 Prognostic indices and a new model

**Figure 1:**
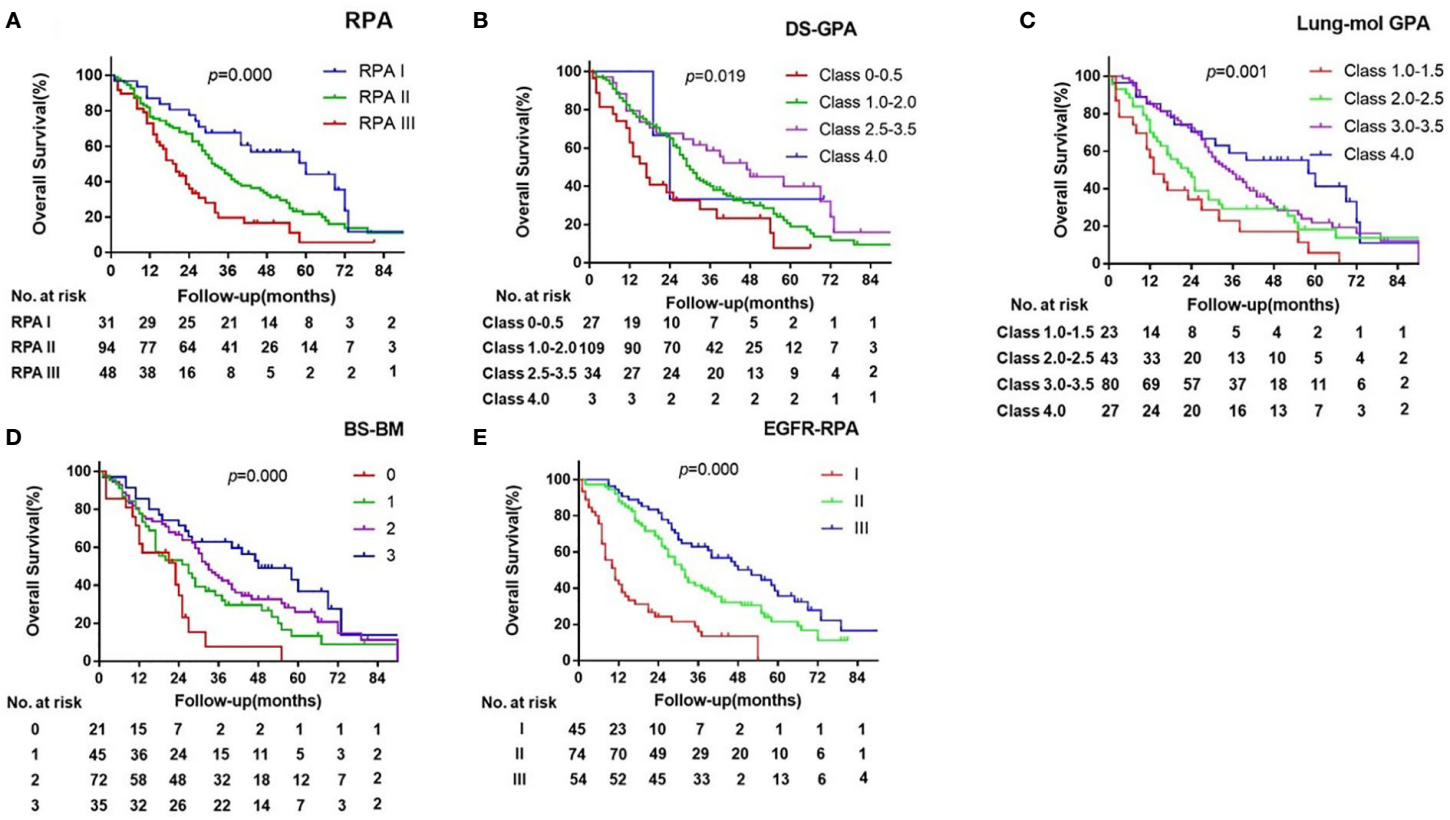
Kaplan-Meier Curves of overall survival showing Survival by the RPA **(A)**, DS-GPA **(B)**, BS-BM **(C)**, lung-mol GPA **(D)** and EGFR-RPA **(E)** for EGFR-mutated lung cancer BM.

**Figure 2:**
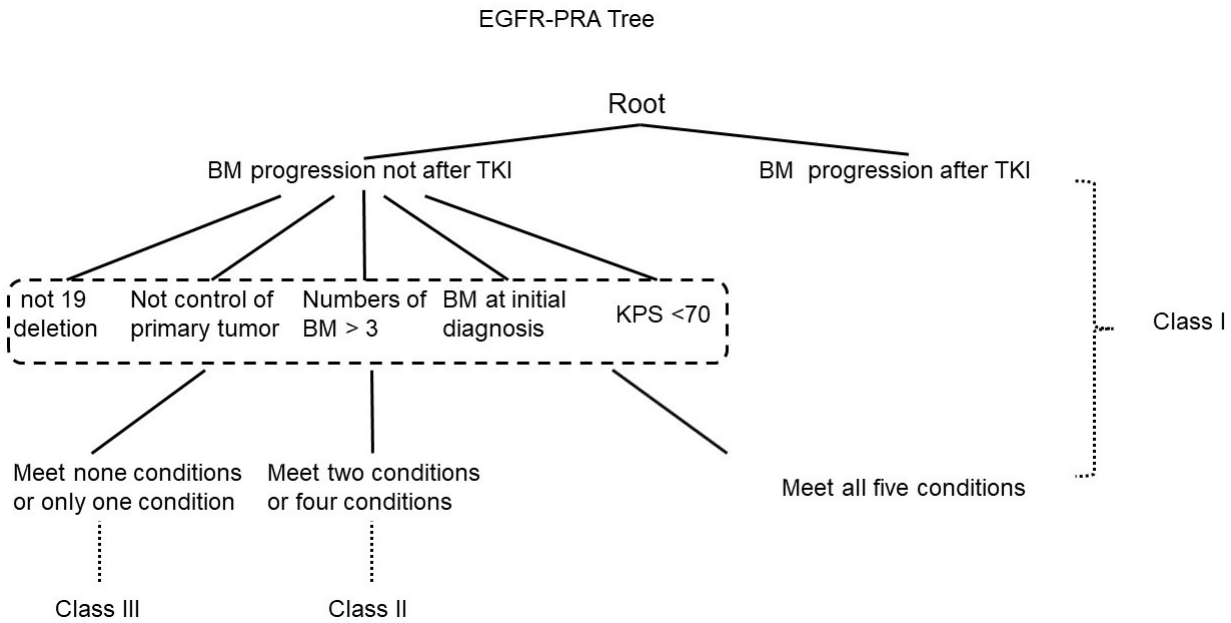
Recursive tree for the new specific prognostic system for epidermal growth factor receptor-mutated lung cancer brain metastases.

The prognostic values of the five indices examined are presented in **Figure 1** and **Supplement Table 1**. In the log-rank test, the indices of RPA, DS-GPA, lung-mol GPA, and BS-BM were all significant predictors of OS. However, they did not demonstrate superiority of their predictive effect. In the multivariate analysis using multiple Cox proportional hazards models, age and extracranial metastases were not found to be the independent prognostic factors for OS. BM at the time of the initial diagnosis of lung cancer (*p=* 0.024), BM progression after TKI (*p=*0.000), and *EGFR* mutation type (*p=*0.023) were independent prognostic factors (**Table 2**); however, they were not associated with the four prognostic indices. Therefore, referring to the RPA model, we established a new BM scoring system named *EGFR*-RPA based on the results of the multivariate analysis (**Figure 2**). The first node split by BM progression after TKI indicated that the survival difference between patients was greater than the difference between any other subset among them. Among the patients with non-TKI advanced BM, the most significant split was the number of prognostic factors. Patients who met 5 prognostic factors or developed BM progression after TKI had the worst survival (Class I). The best survival was observed in patients who had either no or only one prognostic factor (Class III). All the other patients had two to four prognostic factors, forming a middle stage (Class II). The median OS for Class I, Class II, and Class III were 11 months (95% CI, 7-15), 32 months (95% CI, 27-37), and 52 months (95% CI, 34-69), respectively (*p*=0.000 **Figure 1** and **Supplement Table 1**). The 3-year OS rates for Class I, Class II, and Class III were 12%, 40%, and 63%, respectively. The 5-year OS rates for Class I, and Class II, and Class III were 0%, 19%, and 36%, respectively.

### 3.4 Comparison of predictive accuracy for overall survival between prognostic indices

The ROC and C-indices were used to compare the prognostic validity. The AUC values for 1-year OS were 0.565 for RPA (*p*=0.214), 0.752 for DS-GPA (*p*=0.175), 0.641 for lung-mol GPA (*p*=0.007), 0.585 for BS-BM (*p*=0.106), and 0.781 for *EGFR*-RPA (*p*=0.000). The C-indices for the survival probability prediction were 0.603, 0.569, 0.613, 0.595, and 0.671, for each scoring system, respectively. These results suggested that the *EGFR*-RPA model presented with the best AUC values and C-indices (**Tables 3**, **4**). Furthermore, the calibration plot for the probability of 1- year OS presented a good correlation between the *EGFR*-RPA prediction and actual observation. (**Supplement Figure 3**).

**Table 3:** AUC of each scoring model to predict 1 year survival.

Scores	AUC	95% CI	*P* value
RPA	0.565	0.466 - 0.665	0.215
DS-GPA	0.572	0.466 - 0.677	0.174
Lung-mol GPA	0.641	0.538 - 0.744	0.007
BS-BM	0.585	0.483 - 0.687	0.106
EGFR-RPA	0.781	0.693 - 0.868	0.000

AUC, Area under the curve; RPA, Recursive partitioning analysis; DS-GPA, Diagnosis specific graded partitioning analysis; lung-mol GPA, lung-molecular graded prognostic assessment; BS-BM, Basic score for brain metastases; EGFR-RPA, EGFR-recursive partitioning analysis; CI, Confidence interval.

**Table 4:** Predictive value analyses of the 5 scoring systems (C-index).

Score	Classes	No. of patients	C-index
RPA	I/II/III	31/94/48	0.603
DS-GPA	0-0.5/1.0-2.0/2.5-3.5/4.0	27/109/34/3	0.569
Lung-mol GPA	1.0-1.5/2.0-2.5/3.0-3.5/4.0	23/43/80/27	0.613
BS-BM	0/1/2/3	21/45/72/35	0.595
EGFR-RPA	I/II/III	45/74/54	0.671

RPA, Recursive partitioning analysis; DS-GPA, Diagnosis specific graded partitioning analysis; lung-mol GPA, lung-molecular graded prognostic assessment; BS-BM, Basic score for brain metastases; EGFR-RPA, EGFR-recursive partitioning analysis.

## 4 Discussion

To the best of our knowledge, this is the first analysis of patients with *EGFR*-mutated NSCLC who developed BM after receiving all the effective treatments, including first-generation TKIs as first line treatment, Osimertinib as subsequent therapy and brain RT. In this study, we observed that KPS, BM at the time of initial diagnosis, BM progression after TKI, *EGFR* mutation type, uncontrolled primary tumor and the number of BM were the independent prognostic factors for OS in real-world practice. Moreover, our finding confirms that BM progression after TKI presented significantly worse outcome, with a median survival of only 10 months. Therefore, it was necessary to establish a new prognostic index specific for patients with *EGFR*-mutated NSCLC who developed BM and BM progression after TKI should be brought into the index. Compared with the previous models of BM, the new prognostic system (*EGFR*-RPA) can accurately classify or categorize patients according to their prognosis, which can be used to determine optimal and personalized management of patients with *EGFR*-mutated NSCLC who develop BM.

Currently, the scoring systems for BM include RPA, BS-BM, DS-GPA and Lung-mol GPA. The differences between them mainly existed in the selection and management of the prognostic factors. The selection of prognostic factors was based on population differences selected at the time of establishment of each scoring system. KPS plays a decisive role in RPA scoring system. The prognostic factors were equivalent in BS-BM, DS-GPA, and lung-mol GPA, and patient outcomes were stratified by scoring methods. The new model differs from the previous model such that age was not an independent prognostic factor, which is consistent with the results of a study conducted on Chinese patients with BM from *EGFR*-mutated lung cancer (14). We speculated that older patients could tolerate targeted therapy well and, thus, benefit from SRS. Further, Sperduto et al. (15) observed that patients with BM from *EGFR*-mutated NSCLC presented with significantly different survival prognosis with different genetic status, thus, introducing *EGFR* gene mutation status and establishing the lung-mol GPA scoring system. However, the type of *EGFR* mutation was not distinguishable in this system. It has been confirmed that *EGFR*-mutated NSCLC is a genetically heterogeneous disease (16). The most common *EGFR* mutations (exon 19 deletion or L858R mutations) predict sensitivity to *EGFR* TKIs. However, patients with exon 19 deletions demonstrate improvement in OS and progression-free survival (PFS) compared to those harboring the L858R mutations following treatment with first-generation *EGFR* TKIs (17). Additionally, 10% of the patients have an uncommon *EGFR* mutation and are less responsive to *EGFR*-TKI therapy compared to the patients with either of the common mutations (18). In our study, the *EGFR* mutation subtype was an independent predictor for prognosis stratification. Finally, focusing on the *EGFR*-mutated NSCLC BM system, this study observed that BM resistance after TKI, which was not accounted for in the previous BM scoring systems, could identify the patients with the worst outcomes.

In this study, BM progression after TKIs was extremely poor prognostic factor for EGFR-RPA for patients with EGFR mutations.BM progression after TKIs belongs to metachronous BM. However, previous studies (19, 20) and our study have demonstrated that patients with metachronous BM have a better prognosis compared to patients who subsequently develop brain metastases. Most importantly, the other validated prognostic indices, such as KPS, *EGFR* mutation type, control of primary tumor, and the number of BM, were similar between groups with and without BM progression after TKI. The current findings suggest that the poor OS observed in patients with BM after TKI is not secondary to selection bias or differences between patient cohorts, albeit due to the prognostic factor itself. Similarly, Kimberly et al. (21)reported that patients treated with TKI prior to BM diagnosis presented worse outcomes than patients who did not receive targeted therapy prior to BM diagnosis (OS: 9 versus 19.6 months). However, unlike the current study in which the groups included 173 patients and the median follow-up was 67 months, only 54 patients were evaluated with a median follow-up at 8.6 months.

In this study, the second-line treatments for patients with BM progression after TKIs included bevacizumab combined with chemotherapy, Osimertinib targeted therapy, and salvage brain RT. In general, the traditional chemotherapeutic agents used to treat NSCLC do not cross the blood-brain barrier (BBB); Therefore, their effect on CNS metastases is limited (22). Recently, Wu (23) observed that the T790 mutation showed low consistency between cerebrospinal fluid (CSF) and plasma in the study of CSF genotyping in *EGFR*-mutated NSCLC, which could explain the poor response to Osimertinib in patients with T790 mutations detected in plasma. In this study, patients with BM progression after TKIs were treated with salvage RT, and the effect was poor. Performing RT for BM after TKI resistance worsened the occurrence of cerebral radiation necrosis in patients treated with TKIs (24).This may also be one of the reasons for the poor survival rate. Therefore, the presence of BM after TKIs indicates drug resistance, and currently, there is currently a lack of effective treatment.

Despite significant results, our study had limitations. First, the study had the limitations inherent to a retrospective analysis. Second, the potential toxicities associated with RT and their impact on the quality of life were not assessed. Last, all the patients received first- or second-generation *EGFR*-TKIs, but did not receive third-generation TKIs, which have a greater ability to penetrate the BBB than that of first- or second-generation *EGFR* TKIs (25)and could reduce the risk of CNS progression versus standard *EGFR*-TKI *(* 13).

## 5 Conclusions

In conclusion, this study presented that BM progression after TKI and *EGFR* mutation type were specific prognostic factors for *EGFR*-mutated lung cancer BM. The new index, whose ROC and C-index were better than those of previous indices, was more prognostic and divisive than the previous indices. According to the *EGFR*-RPA index, the worst median survival was 10 months, whereas the best median survival was 52 months.

## Data availability statement

The original contributions presented in the study are included in the article/**Supplementary Materials**. Further inquiries can be directed to the corresponding authors.

## Ethics statement

The study was conducted according to the Helsinki Declaration and the study protocol was approved by the Ethics Review Board of the Fudan University Shanghai Cancer Center and the People’s Hospital Affiliated to Jiangsu University. Written informed consent from the patients was not required to participate in this study in accordance with the national legislation and the institutional requirements.

## Author contributions

X-WF provided direction and guidance throughout the preparation of this manuscript. L-HZ wrote and edited the manuscript. L-HZ, LS, T-TN, and Y-QL collected and prepared the related papers. X-WF, L-HZ, K-LW, and C-YW reviewed and made significant revisions to the manuscript. All authors contributed to the article and approved the submitted version.
