# Supplementary material for: New prognostic system specific for epidermal growth factor receptor-mutated lung cancer brain metastasis
Source: Front Oncol. 2023 Mar 20;13:1093084. doi: 10.3389/fonc.2023.1093084 (PMC10067922; doi:10.3389/fonc.2023.1093084)
Supplement: Supplementary file 1 [file Table_1.docx]

**Supplement table 1 Log-rank analyses of the 5 scoring systems**

| **Scores** | **Median OS (months)** | **95% CI** | ***P*** |
| --- | --- | --- | --- |
| RPA |  |  | 0.000 |
| I | 60.0 | 32.044 - 87.956 |  |
| II | 31.0 | 25.338 - 36.662 |  |
| II | 19.0 | 14.274 - 23.726 |  |
| DS-GPA |  |  | 0.019 |
| Class 0-0.5 | 17.0 | 10.252 - 23.748 |  |
| Class 1.0-2.0 | 30.0 | 26.197 - 33.803 |  |
| Class 2.5-3.5 | 47.0 | 32.220 - 61.780 |  |
| Class 4.0 | 24.0 | 15.998 - 32.002 |  |
| Lung-mol GPA |  |  | 0.001 |
| Class 1.0-1.5 | 13.0 | 6.739 - 19.261 |  |
| Class 2.0-2.5 | 23.0 | 16.792 - 29.208 |  |
| Class 3.0-3.5 | 35.0 | 27.537 - 42.463 |  |
| Class 4.0 | 58.0 | 27.762 - 88.238 |  |
| BS-BM |  |  | 0.000 |
| 0 | 23.0 | 9.910 - 36.090 |  |
| 1 | 27.0 | 14.331 - 39.669 |  |
| 2 | 33.0 | 27.544 - 38.456 |  |
| 3 | 48.0 | 28.653 - 67.347 |  |
| EGFR-RPA |  |  | 0.000 |
| I | 11 | 7.080 - 14.920 |  |
| II | 32 | 27.167 - 36.833 |  |
| III | 52 | 34.776 - 69.224 |  |

OS=Overall survival, CI=Confidence interval, RPA=Recursive partitioning analysis, DS-GPA=Diagnosis specific graded partitioning analysis, Lung-mol GPA =Lung-molecular graded prognostic assessment, BS-BM= Basic score for brain metastases.
