# Supplementary figures and images for: New prognostic system specific for epidermal growth factor receptor-mutated lung cancer brain metastasis
Source: Front Oncol. 2023 Mar 20;13:1093084. doi: 10.3389/fonc.2023.1093084 (PMC10067922; doi:10.3389/fonc.2023.1093084)

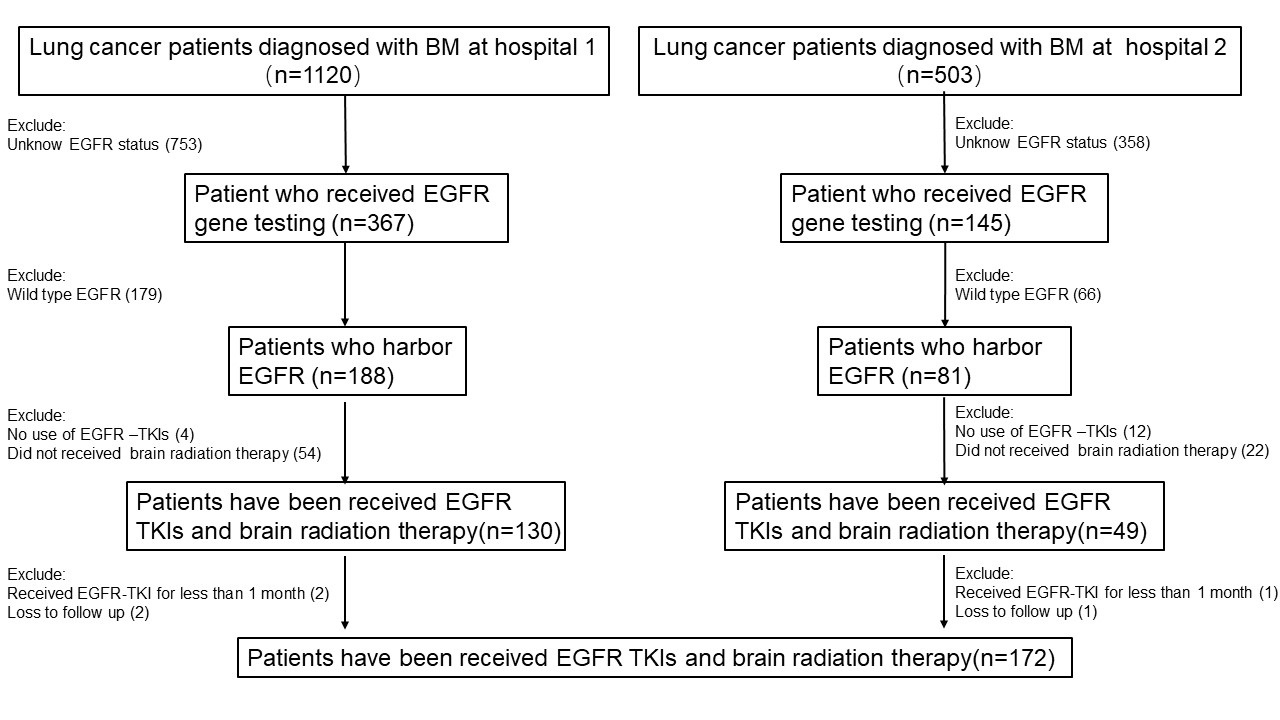

Supplement: Supplementary file 2 [file Image_1.jpeg]

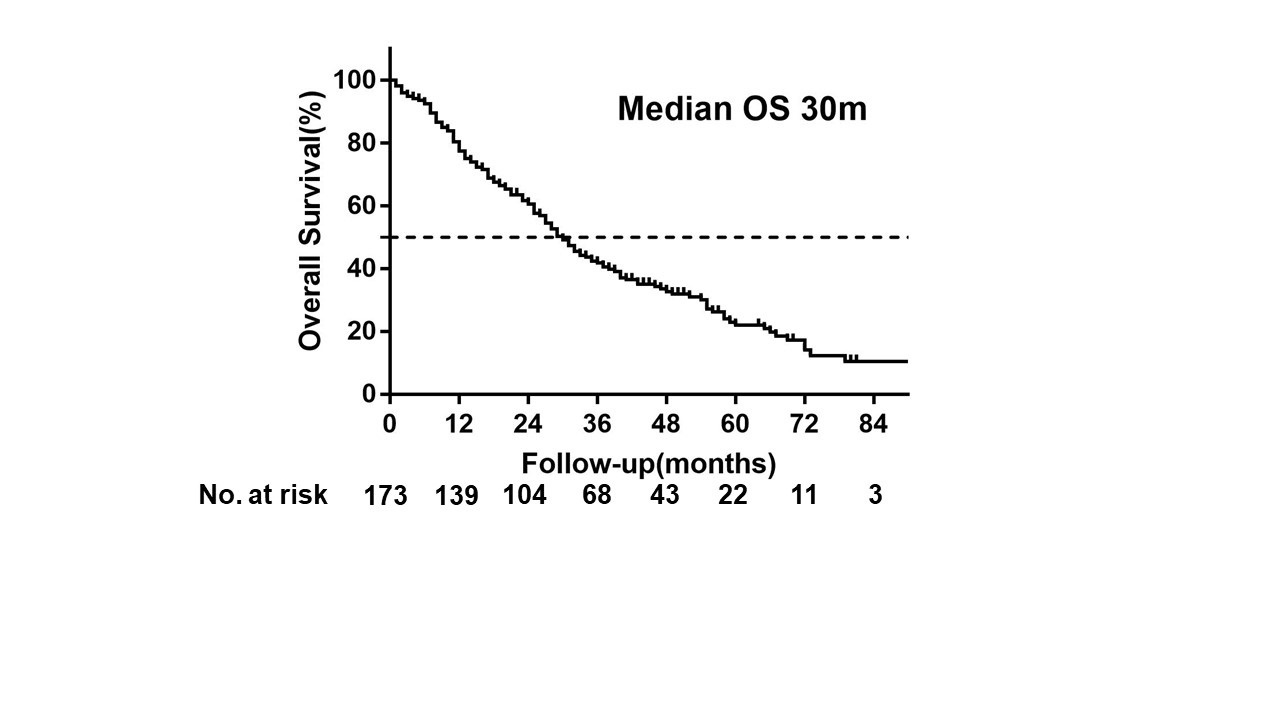

Supplement: Supplementary file 3 [file Image_2.jpeg]

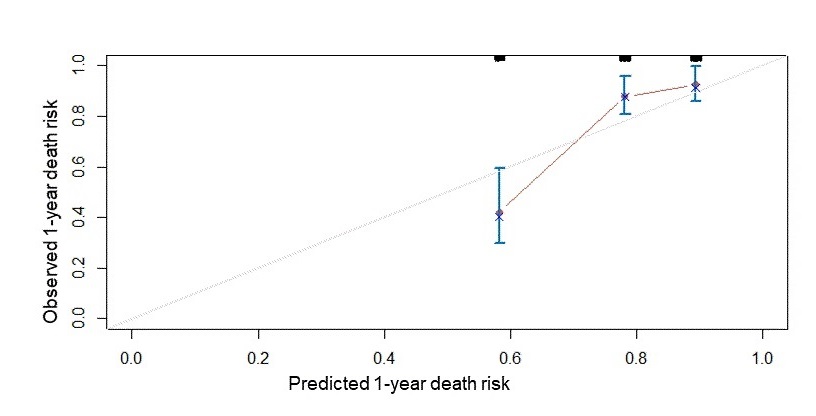

Supplement: Supplementary file 4 [file Image_3.jpeg]
